# Identification of therapeutic targets from genetic association studies using hierarchical component analysis

**DOI:** 10.1186/s13040-020-00216-9

**Published:** 2020-06-17

**Authors:** Hao-Chih Lee, Osamu Ichikawa, Benjamin S. Glicksberg, Aparna A. Divaraniya, Christine E. Becker, Pankaj Agarwal, Joel T. Dudley

**Affiliations:** 1 Department of Genetics and Genomic Sciences, Icahn School of Medicine at Mount Sinai, New York, NY 10029 USA; 2 Institute for Next Generation Healthcare, Icahn School of Medicine at Mount Sinai, New York, NY 10029 USA; 3 Drug Research Division, Sumitomo Dainippon Pharma. Co. Ltd., 3-1-98 Kasugade-naka, Konohana-ku, Osaka, 554-0022 Japan; 4 Hasso Plattner Institute of Digital Health at Mount Sinai, Icahn School of Medicine at Mount Sinai, New York, NY 10032 USA; 5 BioInfi, 1150 First Avenue, Ste 501, King of Prussia, PA 19406 USA

**Keywords:** Genome-wide association study, Network biology, Gene candidate discovery

## Abstract

**Background:**

Mapping disease-associated genetic variants to complex disease pathophysiology is a major challenge in translating findings from genome-wide association studies into novel therapeutic opportunities. The difficulty lies in our limited understanding of how phenotypic traits arise from non-coding genetic variants in highly organized biological systems with heterogeneous gene expression across cells and tissues.

**Results:**

We present a novel strategy, called GWAS component analysis, for transferring disease associations from single-nucleotide polymorphisms to co-expression modules by stacking models trained using reference genome and tissue-specific gene expression data. Application of this method to genome-wide association studies of blood cell counts confirmed that it could detect gene sets enriched in expected cell types. In addition, coupling of our method with Bayesian networks enables GWAS components to be used to discover drug targets.

**Conclusions:**

We tested genome-wide associations of four disease phenotypes, including age-related macular degeneration, Crohn’s disease, ulcerative colitis and rheumatoid arthritis, and demonstrated the proposed method could select more functional genes than S-PrediXcan, the previous single-step model for predicting gene-level associations from SNP-level associations.

## Introduction

Genome-wide association studies (GWAS) seek to identify how genetic variations, typically represented as single-nucleotide polymorphisms (SNPs), contribute to variability in expression of phenotypic traits or diseases across the population. GWAS, which is made possible by the availability of the reference human genome [1, 2], represents contemporary efforts to map collective genetic architecture to common diseases. Since the first GWAS in 2005, applications of this technique have facilitated identification of risk variants for various diseases, including age-related macular degeneration [3], inflammatory bowel disease (IBD) [4–6], type 2 diabetes [7, 8] and many others. For example, GWAS have discovered over 200 risk loci for IBD that encompass a wide range of biological processes, including innate and adaptive immunity, autophagy, and epithelial permeability [9].

Currently, identification of therapeutic targets from GWAS remains difficult and relatively inefficient, largely because SNP associations often do not directly indicate optimal therapeutic targets nor the complex mechanism underlying disease pathogenesis [10]. The presence of non-coding causal SNPs is one of the major obstacles to functional implications of the mechanisms of disease [11]. Studies have demonstrated the widely-spread SNP associations with tiny effect sizes can collectively contribute to a large portion of heritability for complex traits such as schizophrenia [12] and height [13]. These ubiquitous genetic signals across genome, acting directly on any genes, may propagate through interconnected gene regulatory network to affect functions of disease-related genes [14]. Studies have also shown that hub genes, genes interacting with many other genes, are subject to negative evolutionary selection [15–17], hinting the potential of combing network topology with genetic signals in search of therapeutic targets. This “omnigenic” point of view thus make us wonder how to distill the ubiquitous genetic signals into effects on the gene network.

To this end, we developed a hierarchical approach that maps disease associations from SNPs to genes, and eventually to transcriptomic modulation. We first developed tissue-specific co-expression networks to determine co-expression modules, a collection of genes that are modulated concurringly, and used it to demonstrate that genetic associations can be hierarchically mapped to these gene modules. We demonstrate that this approach, requiring only GWAS summary data, determines module associations as accurate as those computed directly from individual-level data. We then applied this technique to GWAS of four complex disorder to demonstrate the applicability of GWAS component analysis and gene candidate discovery.

## Methods

### Overview of the proposed method

**Fig. 1:**
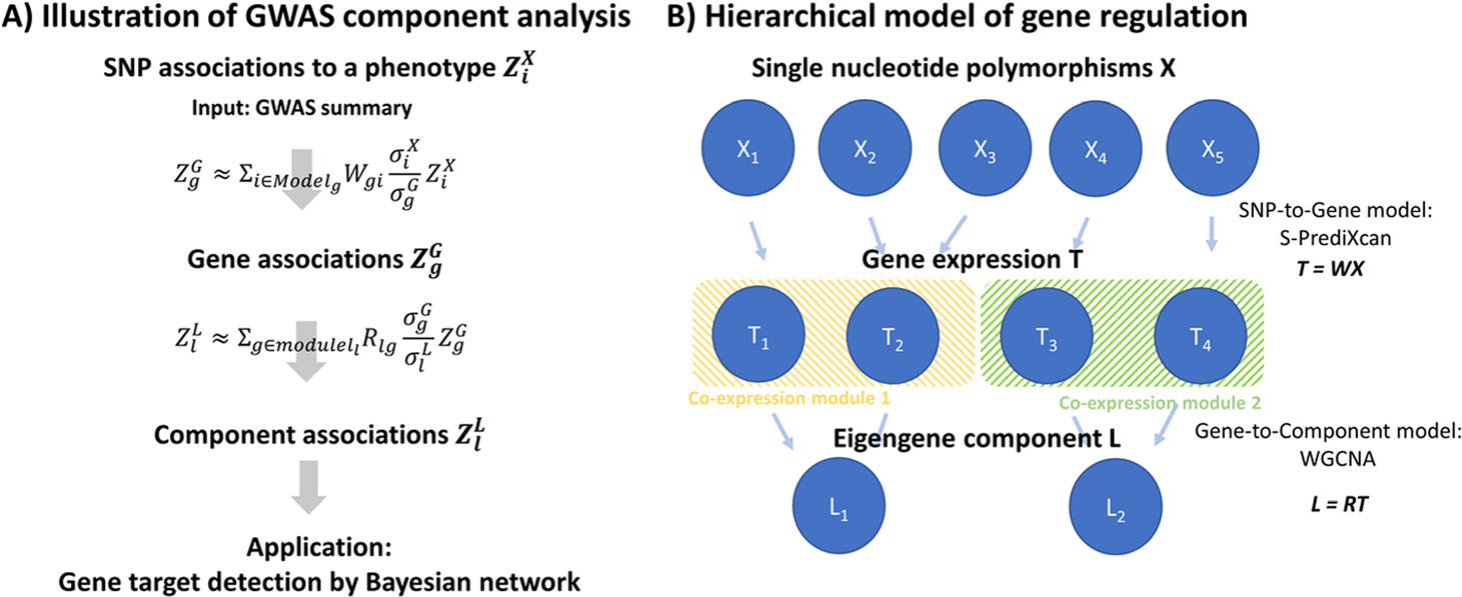
Schematic diagram of GWAS component analysis. **a** We designed a two-stage method to map SNP associations to component associations. Associated GWAS components were integrated with Bayesian networks to facilitate therapeutic discovery. GWAS component is a component with a score ${Z}_l^L$ significantly deviating from 0. **b** In the first stage, S-PrediXcan was used to infer gene associations from SNP associations. Gene-to-Component model were fitted by WGCNA, and the corresponding associations were computed as in Eq. (2) (see text). Models in B) were built using GTEx reference data

We took a two-stage approach to discover disease-associated gene components (Fig. 1a). First, we mapped SNP associations to gene associations using S-PrediXcan [18], which utilizes a linear model that maps SNP dosage to gene expression to predict gene associations ${Z}_g^G$ from SNP associations ${Z}_i^X$ (Fig. 1b). Both associations are linked by

1 $$ {Z}_g^G\approx {\varSigma}_{i\in Mode{l}_g}{W}_{gi}\frac{\sigma_i^X}{\sigma_g^G}{Z}_i^X $$

where *W* is the weight matrix of the linear model fitted using individual-level data from the Genotype–Tissue Expression project (GTEx) [19], and ${\sigma}_g^G$ and ${\sigma}_i^X$ are the standard deviations of a gene *g* and a SNP *i*, respectively.

In the second stage, we estimated the disease association of an eigen-gene component *L*_*l*_ that represents the activity of a co-expression module. A co-expression module represents a group of genes whose expression is collectively modulated, while the eigen-gene component summarizes the overall expression of this gene group by the largest variation. Specifically, the eigengene of a co-expression module is defined as the first principal component of the measured gene expression profile in the module [20]. Given the linearity of principal component analysis, we can compute the eigengene by multiplying a weight matrix *R* to the gene expression matrix. We note that this is analogous to the way S-PrediXcan computes gene expressions from SNP dosages and thus the statistical theory of S-PrediXcan can be carried over, as we summarize in the following. Given the weight matrix *R*, the disease association ${Z}_l^L$ of an eigen-gene component *L*_*l*_ can be estimated by

2 $$ {Z}_l^L\approx {\Sigma}_{g\in Mode{l}_l}{R}_{lg}\frac{\sigma_g^G}{\sigma_l^L}{Z}_g^G, $$

where ${\sigma}_l^L$ is the standard deviation of the eigen-gene component *L*_*l*_. Equations (1) and (2) thus transfer the disease associations from genomic space to transcriptomic space, and ultimately to co-expression modules. Under the null hypothesis, ${Z}_l^L$ is a standard normal random variable. We thus refer to a component, with a score ${Z}_l^L$ significantly deviating from 0 as a genome-wide significant (GWAS) component.

We propose using Bayesian networks (BN) to discover putative causal genes of GWAS components. We sought to discover functional genes by determining the overlap between a GWAS component and a tissue-specific BN. The “functionality” of a gene candidate *g*_0_ is evaluated based on the odds ratio of the overlap between its children on the BN and the GWAS component. Specifically, we selected putative causal genes by testing whether the set *S*_1_ = {*g* ∈ *B*| *g* is in a GWAS component} is overrepresented by *S*_2_ = {*g* ∈ *B*|*g* is downstream of *g*_0_ in the BN}, where *B* is the set of background genes defined by the intersection of genes used in constructing S − PrediXcan models and the Bayesian networks. The Bayesian networks were constructed using RIMBANet [21].

### Computation of gene-level associations by S-PrediXcan

To map SNP associations to gene associations, we used the recently proposed method S-PrediXcan to predict tissue-specific gene associations. We briefly summarize S-PrediXcan as follows: given *X*_*i*_, the allelic dosage for SNP *i*, *T*_*g*_, the predicted expression of gene *g*, and *Y*, the phenotype of interest, S-PrediXcan assumes a pre-trained model that maps allelic dosages to the predicted expression by

3 $$ {T}_g={\Sigma}_{i\in Mode{l}_g}{W}_{gi}{X}_i+\epsilon $$

where *W* is the weight matrix of the linear model fitted using individual-level genotype data [18]. On top of this linear model, S-PrediXcan estimate the gene association ${Z}_g^G={\gamma}_g/ se\left({\gamma}_g\right)$ from the SNP associations ${Z}_i^X={\beta}_i/ se\left({\beta}_i\right)$, where *β*_*i*_ and *γ*_*g*_ are estimators of effect sizes and *se*(*β*_*i*_) and *se*(*γ*_*g*_) are the variances of the estimators of gene *g* and SNP *i*, respectively. Barbeira et al. [18] demonstrated that both associations are approximately related by

4 $$ {Z}_g^G\approx {\Sigma}_{i\in Mode{l}_g}{W}_{gi}\frac{\sigma_i^X}{\sigma_g^G}{Z}_i^X $$

where ${\sigma}_g^G$ and ${\sigma}_i^X$ are the standard deviations of gene *g* and SNP *l*. Similar results were obtained via a different derivation [22]. We summarized their approximation as follows: Given random variables *X*_*i*_ whose covariance *Γ* is known, the association of its linear transformation ${T}_g={\Sigma}_{i\in Mode{l}_g}{W}_{gi}{X}_i$ to trait *Y* can be approximated by Eq. (4), where ${\sigma}_g^G={\sum}_{ij}{W}_{ig}{\Gamma}_{ij}{W}_{gj}$.

Similar methods exist for mapping SNP associations to gene associations. Several methods infer gene-level associations as aggregated effects of a group of SNPs by modeling the linkage disequilibrium (LD) structure using, for example, chi-squared statistics [23, 24] or hypothesis testing [25]. Another class of methods attempt to integrate expression quantitative trait loci (eQTLs) with GWAS signals. For example, COLOC seeks to determine whether eQTL and GWAS signals are consistent with a shared causal variant [26]. Summary mendelian randomization (SMR) includes instrumental variables to determine the causative effects of gene expressions on a phenotype from eQTLs [27]. TWAS [22] and S-PrediXcan [18] combine information of the LD structure and eQTLs into multivariate analysis to infer trait-associated genes. Theoretical and empirical comparison on COLOC, SMR, TWAS and S-PrediXcan can be found in [18].

### Computation of GWAS component associations

Our proposed method further assumes that overall activity of a co-expression module, termed eigen-gene component *L*, can be represented by a mixture of gene expression *T*, i.e.,

$$ {L}_l={\varSigma}_{g\in Modul{e}_l}{R}_{lg}{T}_g. $$

The matrix *R* consists of the weighted contributions of genes to an eigen-gene component. Applying the relation in Eq. (4) to *L*, trait association ${Z}_l^L$ can be approximated by

$$ {Z}_l^L\approx {\varSigma}_{g\in Modul{e}_l}{R}_{lg}\frac{\sigma_g^G}{\sigma_l^L}{Z}_g^G $$

### Building the eigen-gene component models

To determine the weight matrix *R*, we applied weighted correlation network analysis (WGCNA) to the GTEx RNA-seq data. Covariates were first removed following the procedure used in building S-PrediXcan models. For consistency, we confined the analysis to the same genes used in building S-PrediXcan models. Co-expression modules were estimated from each tissue independently. We tuned the minimum of module size to 5 to allow detection smaller modules. The eigen-gene component was then computed as the first principal component of the expression matrix of co-expressed genes.

### Construction of Bayesian networks

The Bayesian networks (BN) were constructed using RIMBANet [21, 28, 29]. The estimation and validation of BNs are reported in previous studies [30, 31]. Briefly, GTEx data were first normalized to ensure a normal distribution, and then discretized into three clusters using the *k*-means approach. The number of clusters was adjusted to two if any of the three clusters contained only a few samples. Each gene was limited to having no more than three parent nodes. The final network was pooled into a consensus network from 1000 repeated runs. Cycles and weak edges were then pruned to ensure that the final network was a directed acyclic graph.

### Simulation test

We simulated a scenario to test Eq. (1) using genotype data of 2504 individuals from the 1000 Genomes Project [32]. We first used S-PrediXcan to compute the predicted gene expression of these 2504 individuals. Eigen-gene components were then computed as weighted averages of these predicted gene expression using WGCNA models fitted from the GTEx Whole Blood data. We then simulated a trait caused by a single component as *Y* = *L*_1_ + *α ϵ* with a randomly selected eigen-gene component *L*_1_. We tested the GWAS component method under various signal-to-noise ratios (SNRs) *std*(*L*_1_)/(*std*(*L*_1_) + *α*), which represents heritability in a broad sense. The selected component is referred to as the causal component, whereas the other components are non-causal. In this scenario, we expect to see a strong z-score from the selected component and minor signals from the other components. The associations to the eigen-gene components were then tested using 1) predicted eigen-gene components from genotypes of 2504 individuals and 2) the proposed GWAS component analysis.

### In silico validations of putative targets

To evaluate these gene candidates, we conducted two in silico validations. First, we evaluated whether mutations in gene candidates could result in disease phenotypes in mouse models. The Phenotype/Alleles project of Mouse Genotype Informatics (MGI) is a database that provides rich information about mutant alleles and their resultant phenotypes in various mouse models [33]. We extracted the phenotypes of disease mouse models (Supplementary Table 6) from MGI. Phenotypes associated with gene candidates were also obtained from MGI. We considered a gene candidate to be a hit if its associated phenotypes were significantly enriched in the phenotypes of at least one disease mouse model.

Second, we evaluated whether the perturbation of a gene candidate could result in disease signatures in cell lines. For this purpose, we queried the characteristic direction of a single-gene perturbation, including shRNA knockdown, overexpression and ligand binding, from L1000CDS [34]. Disease characteristic direction signatures were constructed using crowd curated data CREED [35], including one AMD, five CD, 22 UC and seven RA case–control studies (Supplementary Table 7). The cosine distance was used to evaluate the relevance of two characteristic direction signatures. Specifically, a gene candidate was considered a LINCS hit if its characteristic direction was significantly correlated (cosine distance ~ 1) or anticorrelated (cosine distance ~ − 1) with at least one disease characteristic direction.

### Statistical overrepresentation test

The overrepresentation test is a statistical test for determining whether the level of overlap between two sets is due to chance. The test requires three inputs: two sets *S*_1_, *S*_2_ to be compared and a background set *B*. It is assumed that the elements in *S*_1_ and *S*_2_ are all drawn from the background set *B*. The chance that the observed data were generated by random overlap can be evaluated by the hypergeometric distribution

$$ p=\frac{\left(a+b\right)!\left(c+d\right)!\left(a+c\right)!\left(b+d\right)!}{n!a!b!c!d!} $$

where

$$ a=\#\left({S}_1\cap {S}_2\right),b=\#\left({S}_1\setminus {S}_2\right),c=\#\left({S}_2\setminus {S}_1\right),d=\#\left(B\setminus \left({S}_1\bigcup {S}_2\right)\right) $$

are elements of the contingency matrix. For significant associations between *S*_1_ and *S*_2_, we define enrichment odds ratio *OR = (a/b)/(c/d).* Two sets are said to be enriched if *OR* is greater than *1* and *p* is less than a given threshold.

We summarize the overrepresentation tests used in our study below:

1. Bayesian network: in the results section, we sought to discover functional genes by determining the overlap between an associated component and a tissue-specific BN. The “functionality” of a gene candidate *g*_0_ is evaluated based on the OR of the overlap between its children and the associated components. Specifically, the inputs for the overrepresentation test are as follows:
  $$ B=\left\{\mathrm{genes}\ \mathrm{used}\ \mathrm{in}\ \mathrm{S}-\mathrm{PrediXcan}\right\}\bigcap \left\{\mathrm{genes}\ \mathrm{in}\ \mathrm{the}\ \mathrm{Bayesian}\ \mathrm{Network}\right\} $$
  $$ {S}_1=\left\{g\in B|g\ \mathrm{is}\ \mathrm{in}\ \mathrm{an}\ \mathrm{GWAS}\ \mathrm{component}\right\} $$
  $$ {S}_2=\left\{g\in B\right|g\ i\mathrm{s}\ \mathrm{downstream}\ \mathrm{of}\ {g}_0\ \mathrm{in}\ \mathrm{the}\ \mathrm{Bayesian}\ \mathrm{Network}\Big\} $$
2. Mouse genome informatics: in the results section, we evaluated in silico whether the mutation of a gene has been associated with relevant disease phenotypes in a mouse model. We set up the overrepresentation test as follows
  $$ B=\left\{\mathrm{all}\ \mathrm{mouse}\ \mathrm{phenotypes}\ \mathrm{in}\ \mathrm{MGI}\right\} $$
  $$ {S}_1=\left\{p\in B|p\ \mathrm{is}\ \mathrm{a}\ \mathrm{phenotype}\ \mathrm{associated}\ \mathrm{with}\ \mathrm{a}\ \mathrm{gene}\ \mathrm{candidate}\right\} $$
  $$ {S}_2=\left\{p\in B\right|p\ \mathrm{is}\ \mathrm{a}\ \mathrm{phenotype}\ \mathrm{of}\ \mathrm{the}\ \mathrm{mouse}\ \mathrm{model}\Big\} $$

### Matching characteristic directions

The characteristic direction is a computational method for finding a high-dimensional vector that best differentiates gene expression between cases and controls [36]. The characteristic direction, generally unit-normalized, is determined as the maximizer of the ratio of posteriors of two Gaussians with a shared covariance:

$$ \mathit{\log}\frac{P\left(G=0|X=x\right)}{P\left(G=1|X=x\right)}=\log \frac{\pi_0}{\pi_1}-\frac{1}{2}{\left({\mu}_0-{\mu}_1\right)}^T{\Sigma}^{-1}\left({\mu}_0-{\mu}_1\right)+{x}^T{\Sigma}^{-1}\left({\mu}_0-{\mu}_1\right). $$

*G* indicates the cases and controls. We aim to compare how similar the characteristic direction of a gene perturbation experiment is to the characteristic direction of a disease. We followed Clark. et al. to compute the similarity of two characteristic directions by the cosine distance:

$$ d\left({v}_1,{v}_2\right)=\left\langle {v}_1,{v}_2\right\rangle /\left|\left|{v}_1\right|\right|\left\Vert {v}_2\right\Vert $$

To estimate the null distribution, we randomly sampled 10,000 characteristic directions and computed the cosine distance between sampled characteristic directions and the targeted disease characteristic direction. We found the empirical distribution is roughly bell-shape but slightly skewed. We thus used the average and standard deviation of this empirical distribution to normalize cosine distance. We call a LINCS hit if the absolute value of the normalized cosine distance from a given gene-perturbed characteristic direction to a target disease characteristic direction is larger than 1.96.

### Multiple testing correction

We used the Holm-Sidak method to correct the family-wise error rate when required. Specifically:

1. When testing the association of disease and component we corrected the number of components tested in each tissue.
2. The LINCS database contains replicates of single-gene perturbation across cell type and time points. The CREEDS database also contains replicates of disease signatures. We thus corrected for the number of combinations of disease signatures and gene perturbation signatures when testing whether perturbation of a gene candidate could result in producing disease signatures in cell lines.
3. The MGI database may contain several mouse models of the same disease. We corrected the number of mouse models of each disease when testing whether the mutation of a gene has been associated with relevant disease in a mouse model.

## Results

**Fig. 2:**
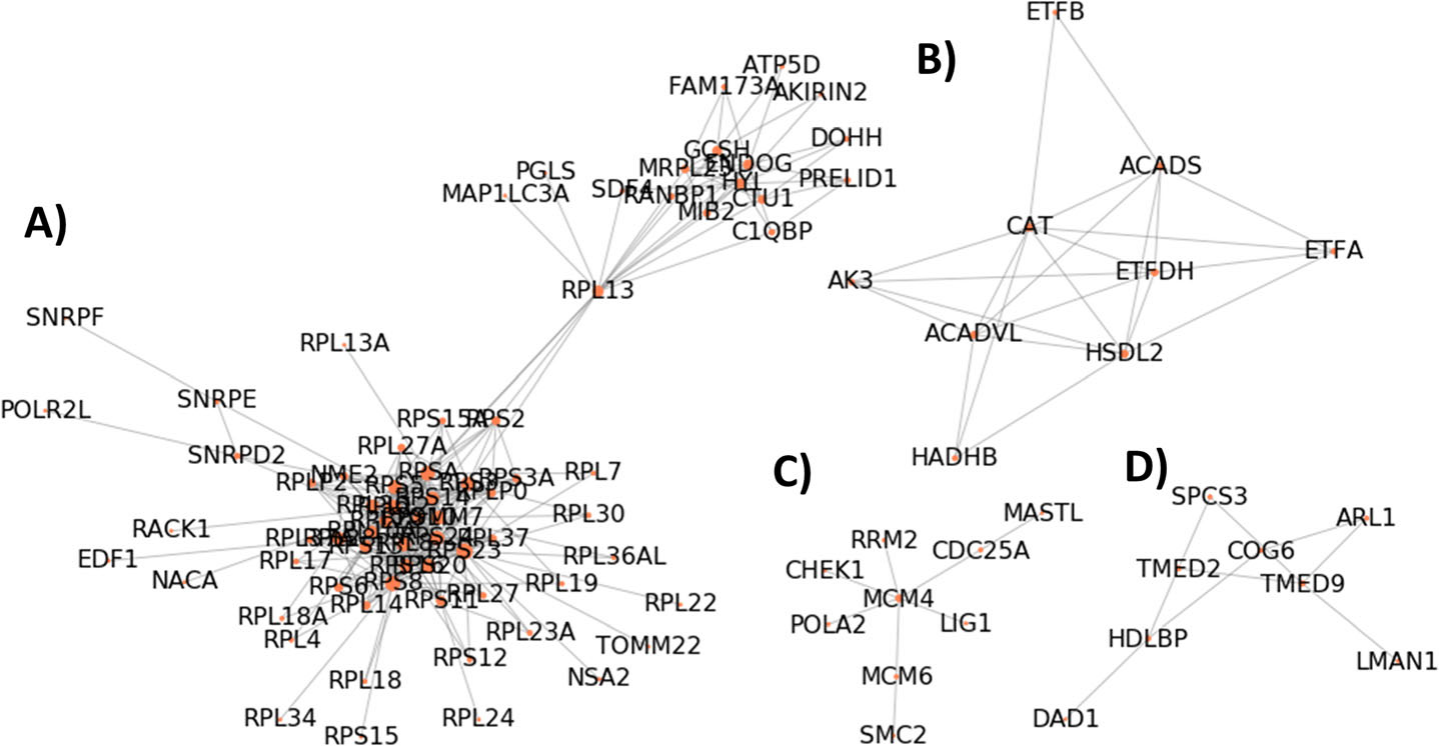
Connected components formed by gene-pairs co-expressed in more than 2 tissues have well-defined biological functions, such as **a** ribosome complex, **b** electron transfer chain, **c** mini-chromosome maintenance and **d** Golgi vesicle transport

To determine the weight matrix *R*, we applied weighted correlation network analysis (WGCNA) to RNA-seq data from GTEx to infer co-expression modules (Methods). Among 44 tissues analyzed, we generally detected 213 ± 89 co-expression modules in one tissue. On average, each component contains 19 genes. In general, the co-expression modules determined by WGCNA are likely to reflect biological pathways and gene functions [20], and we sought to probe if these co-expression modules were linked to genetics. We compared the co-expression modules to a multi-species co-expression network, in which the gene-gene interactions are present in multiple species and assumed to be genetically conserved [37]. Specifically, we formed a network by enumerating all gene-gene combinations within WGCNA modules and compare it to the multi-species co-expression network. We found that, for a single tissue, ~ 2% WGCNA edges are overlapped with the multi-species co-expression network despite the overlap is very significant (Odds ratios range from 1.45 to 39.87, Supplementary Table 1). In addition, 23% WGCNA edges, if detected in more than 2 tissues, can be found in the multi-species co-expression network (Table 1). We also found that connected components formed by WGCNA edges detected in more than 2 tissues carry clearly defined biological functions, such as ribosomal protein synthesis, ATP synthesis and structural maintenance of chromosomes (Fig. 2a-d). Overall, these results show that co-expression models estimated by WGCNA are consistent with biological knowledge.

**Table 1:** Gene pairs co-expressed in multiple tissues and genetically conserved gene pairs. P-values report the difference in ratios compared to the one estimated from gene pairs found in one tissue (bottom row)

# tissues detected	# WGCNA edge (n_1_)	# genetically conserved edges (n_2_)	ratio (n_2_ / n_1_)	*p*-value
14	1	0	0	0.548
13	1	1	1	8.78 × 10^−23^
12	7	2	0.286	3.58 × 10^−13^
11	9	5	0.556	1.68 × 10^−58^
10	12	4	0.333	1.75 × 10^−28^
9	19	8	0.420	2.06 × 10^− 69^
8	28	15	0.536	6.96 × 10^− 163^
7	33	14	0.424	6.65 × 10^− 120^
6	43	14	0.326	3.58 × 10^− 91^
5	75	20	0.267	4.62 × 10^− 104^
4	180	45	0.25	1.18 × 10^− 209^
3	495	87	0.176	2.21 × 10^− 261^
2	3344	218	0.0652	1.96 × 10^− 170^
1	78,656	817	0.0104	N/A

**Fig. 3:**
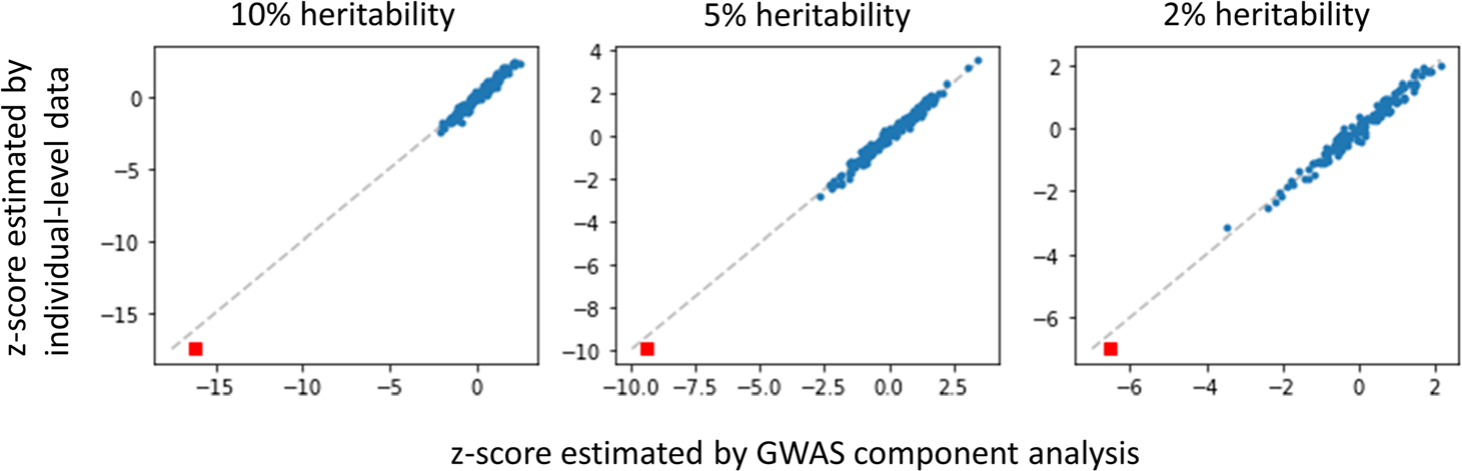
Component associations estimated using GWAS summary (x-axis) are consistent with estimated using individual-level data (y-axis). Data were simulated as a trait that can be attributed to a randomly chosen eigen-gene component with 10% (left), 5% (middle) and 2% (right) heritability. Red squares indicate the z-score of the causal component. We confirmed by Kolmogorov–Smirnov test that the z-scores from non-causal components (blue points) followed a normal distribution (*p* = 0.1691, 0.5393 and 0.2542 for 10, 5 and 2% (right) heritability, respectively)

We first validated that the associations estimated by Eq. (2) using summary-level data is consistent with those estimated using individual-level data. To this end, we simulated gene expressions and the eigen-gene activity from individual genotype data using PrediXcan [38]. 2504 samples were simulated using the genotype data collected in the 1000 Genomes Project [32]. We then randomly selected an eigen-gene component and used its activity, injected with different level of random noises, as a trait to conduct a Genome-wide association study. The summary statistics of these SNP correlations was used as input to compute component associations using Eqs. (1) and (2) and benchmark against the associations estimated directly using simulated eigen-gene activity. The results showed a linear correspondence between associations estimated by individual-level and summary data (Fig. 3). We confirmed by Kolmogorov–Smirnov test that the z-scores from non-causal components followed a normal distribution. These results show that the proposed method conforms the statistical theory of S-PrediXcan.

**Fig. 4:**
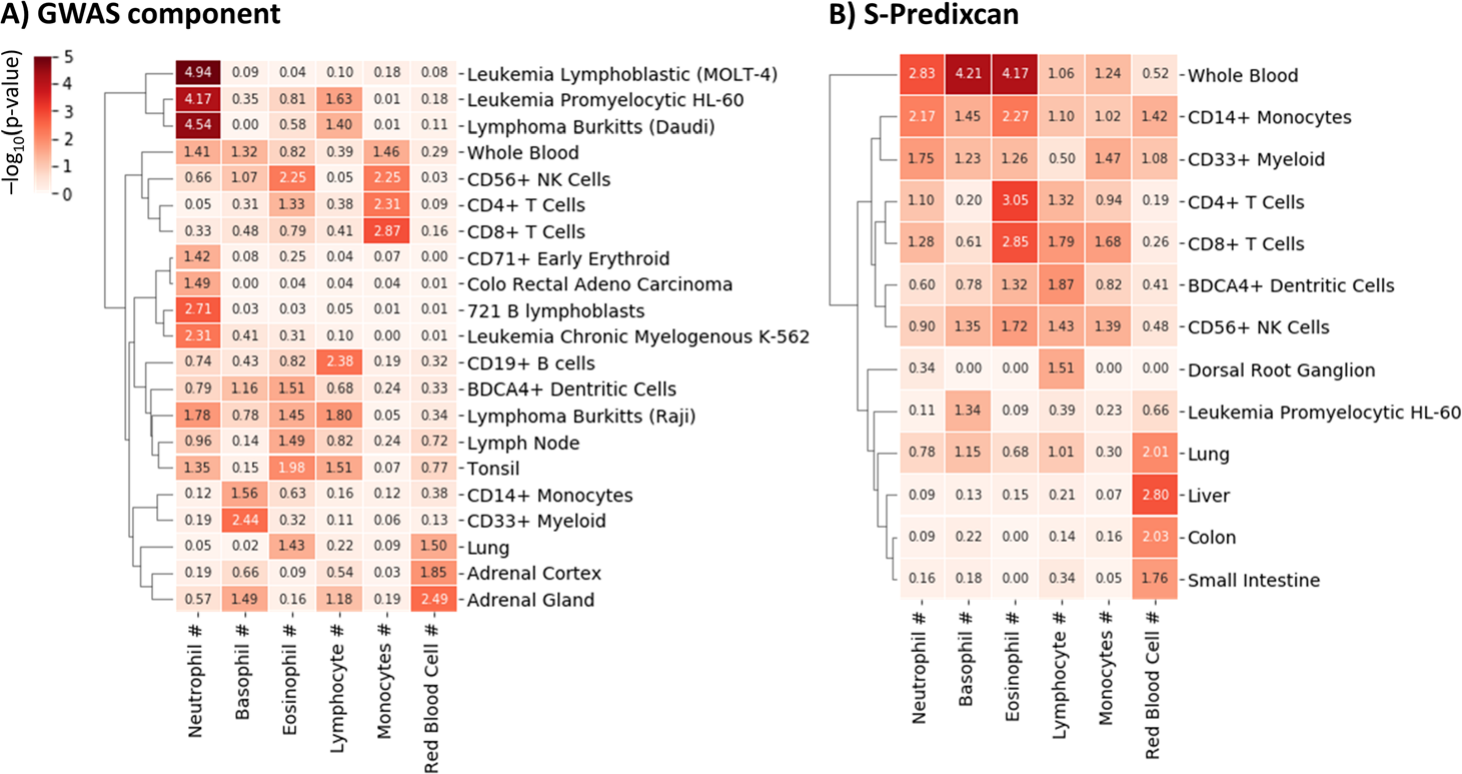
GWAS components select cell-specific signatures. Cell type enrichment were used to determine cell types from genes selected by GWAS component analysis (**a**) and S-Predixcan (**b**). Colors indicate –log_10_(p-value). Results were generated by CTen web server

Next, we investigated whether the associated components capture biological information. We applied our method to GWAS summary statistics of six traits of blood cell counts [39], including neutrophils, eosinophils, basophils, lymphocytes, monocytes and red blood cells, to obtain GWAS components of these six traits in the whole blood tissue. The whole blood tissue was selected since all 6 GWAS traits are measured from blood samples. We then performed cell type enrichment analysis [40] to determine the relevant cell types using genes from GWAS components. Figure 4 shows the p-values of the enriched cell types. Genes of GWAS components associated with lymphocyte counts, basophil counts, and neutrophil counts are enriched in B cells, Myeloid cells, and the neutrophil-like HL-60 cell line respectively. These results confirmed that our method could capture gene sets enriched in expected cell types, though we did not observe a perfect one-to-one correspondence.

Last, we investigated the potential of our approach to discover putative therapeutic gene targets. To this end, it would be valuable to discover targets that might specifically impact the gene component. We projected the associated components onto Bayesian networks (BNs) constructed from GTEx [31] data. We ranked BN genes by the odds ratio of overlap between a node’s downstream genes and genes in the GWAS component. Significance was determined by testing whether the odds ratio is statistically greater than 1. We applied this approach to four disease phenotypes and discovered 147, 47, 103 and 158 putative gene targets for age-related macular degeneration (AMD), Crohn’s disease (CD), ulcerative colitis (UC) and rheumatoid arthritis (RA) respectively. The full list of significant gene targets is provided in Supplementary Tables 2, 3, 4, 5.

With the “omnigenic” point of view [14], we wonder if “core genes” on the gene regulatory network are better therapeutic targets than “peripheral genes” that directly carry genetic variations. Core genes are defined as functional genes that give rise to phenotypes but are not necessarily carrying genetic variants. Our approach attempts to capture this subset of genes while we used S-PrediXcan’s results to represent “peripheral genes” related directly to genetic variations. MGI hits and LINCS hits are used to measure the possibility of a gene being a therapeutic target. In Table 2, we reported the ratio of functional genes among all gene candidates available in each database. Overall, we demonstrated the proposed methods could select more functional genes than S-PrediXcan that selects genes directly influenced by SNP-level associations. Especially, we observed a higher ratio of MGI hits, but a comparable rate in LINCS hits, using GWAS components. Among these gene candidates, 5 of them were targets of known medications (Table 3) listed on DrugBank. TNF, selected by S-PrediXcan from both UC and AMD GWAS summary, is a target of infliximab, Chloroquine and Etanercept. ALOX5, selected by our approach as a gene candidate for UC, is a target of Mesalazine. SLOC1A2, FCGR3A and C1QA, selected by our approach as gene candidates for RA, are also targets of medications for RA such as Etanercept, Hydrocortisone and Ibuprofen. These results further support the idea that our approach can improve selection of functional gene candidates from a GWAS summary.

**Table 2:** In silico validation of gene candidates for four disease phenotypes

	AMD	CD	UC	RA
**GWAS components + BNs (*p***_***1***_**)**				
LINCS hits	0.059 (3/51)	0.143 (2/14)	0.146 (6/41)	0.326 (17/52)
MGI hits	0.311 (42/135)	0.326 (15/46)	0.361 (35/97)	0.493 (73/148)
**S-Predixcan (*p***_***2***_**)**				
LINCS hits	0.093 (5/54)	0.243 (18/74)	0.121 (7/58)	0.237 (22/93)
MGI hits	0.227 (29/128)	0.367 (50/136)	0.269 (28/104)	0.277 (57/206)
***p***_***1***_***– p***_***2***_				
LINCS hits	−0.034	−0.1	0.025	0.089
MGI hits	0.084	− 0.047	0.092	0.216^***^

Numerators represent hits, and denominators represent the number of genes retrieved by GWAS components + BNs or S-PrediXcan. *** indicates *p* < 0.001

**Table 3:** Gene candidates with known indications. Results are queried from DrugBank

Target	Indication	Drug
**GWAS components + BNs**		
ALOX5	UC	Mesalazine
SLOC1A2	RA	Hydrocortisone, Ibuprofen, Indomethacin
FCGR3A	RA	Etanercept
C1QA	RA	Etanercept
**S-PrediXcan**		
TNF	UC	Infliximab
TNF	RA	Chloroquine, Etanercept, Infliximab

## Discussion

**Fig. 5:**
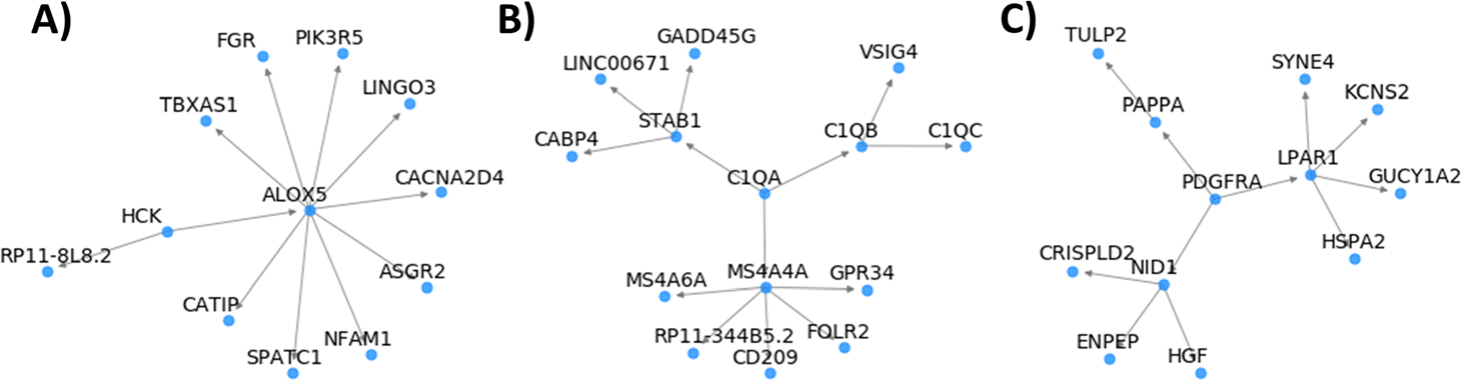
Downstream genes of selected gene candidates on the Bayesian networks: **a** HCK in artery aorta, **b** C1QA in adrenal gland, and **c** PDGFRA in adrenal gland. For simplicity, genes more than three steps away from a gene candidate were excluded

We investigated a few genes captured by our approach and are both MGI and LINCS hits. HCK was found to be a gene candidate driving a GWAS component associated with UC in artery aorta. HCK was previously found to be genetically associated with inflammatory bowel disease and predicted as a causal factor that regulates NOD2, IL10 and ALOX5 [41]. Consistent with this, the BN suggests that HCK regulates ALOX5 (Fig. 5a), whose absence has a protective role in an experimental mouse model of colitis [42].

C1QA was found to be a gene candidate from an AMD-associated component in adrenal gland. C1q and the classical complement pathway has been suspected to play a role in the disease progression induced in retinal degeneration, potentially through local expression of C1q from subretinal microglia/macrophages that instigates inflammasome activation and inflammation [43]. Inspection of the neighborhood on the BN suggests that C1QA regulates MS4A4A (Fig. 5b), a membrane-spanning protein that is expressed on macrophage-lineage cells [44, 45].

We also identified PDGFRA as a gene candidate from a RA-associated component in stomach. PDGFR has been found to be upregulated in RA synoviocytes and synovial tissues and may play a role in synoviocyte-driven extracellular matrix degradation in RA [46]. PDGFR signaling has been shown to be one of potential mechanisms of immatinib mesylate, a tyrosine kinase inhibitor that reduces activation of RA synoviocytes [47]. Inspection of the neighborhood on the BN (Fig. 5c) suggests that PDGFRA regulate LPAR1 which may contribute to development of arthritis via cellular infiltration [48].

GWAS component analysis provides a complementary viewpoint to genetic mapping. Instead of locating risk variants, this approach looks for transcriptomic modulation that is influenced by genetic variants. This added dimension allows interpretation of GWAS results on pathways more relevant to phenotypes. In contrast to previously developed techniques, our method detects novel disease-associated components rather than enriched pathways from databases [49]. In this study, we applied WGCNA to single-tissue gene expression independently. As our results showed that genes co-expressed in multiple tissues usually carry well-defined functions, integrating multiple tissues may improve the construction of co-expression networks, as has been done previously [50]. However, such joint modeling often operates on shared genes across tissues, limiting its applicability when integrating with S-Predixcan models. Currently our method utilizes WGCNA to estimate co-expression modules in an unsupervised manner. Further work is required to integrate WGCNA with GWAS summary to construct a disease centric co-expression network.

The key to GWAS component analysis is its ability to utilize and stack models estimated from the reference genome and tissue-specific gene expression in a principled way. Combining models is crucial to obtaining a holistic picture of the complex biological systems underlying diseases [51, 52]. Although comprehensive measurements of every aspect of these systems would in theory offer a direct solution, such data are generally lacking. Instead, reference data focused on specific features of systems are accumulating at unprecedented speed. In this study, we combined two models in sequential order, demonstrating the feasibility of combining co-expression networks with GWAS associations. In the future, we expect to integrate additional types of functional data into this framework, and we envision that general approach of combining local models estimated from various data sources will enable comprehensive characterization of complex diseases.

## Conclusions

Here we describe a hierarchical approach, GWAS component analysis, for detecting disease-associated components from GWAS summary data. GWAS component analysis utilizes correlations of gene expression to further summarize SNP associations into associations of eigen-gene components. We evaluated GWAS component analysis on synthetic data and confirmed its consistency with respect to associations estimated using individual-level data. The application to GWAS of six blood cell counts revealed enriched cell types that coincide with current knowledge. We further demonstrated that GWAS component analysis can be used for therapeutics discovery by coupling it with Bayesian networks. Investigation of selected gene candidates suggests that our integrated framework can discover functional gene candidates from a GWAS summary.

## Supplementary information

**Additional file 1.**

**Additional file 2.**

**Additional file 3.**

**Additional file 4.**

**Additional file 5.**

**Additional file 6.**

**Additional file 7.**

## Data Availability

Python scripts for reproducing results are available on https://github.com/howchihlee/gwas_component_analysis.

## References

[1] Venter JC, Adams MD, Myers EW, Li PW, Mural RJ, Sutton GG (2001). The sequence of the human genome. Science (80- ).

[2] Consortium†The International HapMap (2003). The International HapMap Project. Nature.

[3] Haines JL, Hauser MA, Schmidt S, Scott WK, Olson LM, Gallins P (2005). Complement factor H variant increases the risk of age-related macular degeneration. Science (80- ).

[4] Duerr RH, Taylor KD, Brant SR, Rioux JD, Silverberg MS, Daly MJ (2006). A genome-wide association study identifies IL23R as an inflammatory bowel disease gene. Science (80- ).

[5] Rioux JD, Xavier RJ, Taylor KD, Silverberg MS, Goyette P, Huett A (2007). Genome-wide association study identifies five novel susceptibility loci for Crohn’s disease and implicates a role for autophagy in disease pathogenesis. Nat Genet.

[6] Liu JZ, van Sommeren S, Huang H, Ng SC, Alberts R, Takahashi A (2015). Association analyses identify 38 susceptibility loci for inflammatory bowel disease and highlight shared genetic risk across populations. Nat Genet.

[7] Demenais F, Kanninen T, Lindgren CM, Wiltshire S, Gaget S, Dandrieux C (2003). A meta-analysis of four European genome screens (GIFT consortium) shows evidence for a novel region on chromosome 17p11. 2–q22 linked to type 2 diabetes. Hum Mol Genet.

[8] Guan W, Pluzhnikov A, Cox NJ, Boehnke M (2008). Meta-analysis of 23 type 2 diabetes linkage studies from the international type 2 diabetes linkage analysis consortium. Hum Hered.

[9] Van Limbergen J, Wilson DC, Satsangi J (2009). The genetics of Crohn’s disease. Annu Rev Genomics Hum Genet.

[10] Timpson NJ, Greenwood CMT, Soranzo N, Lawson DJ, Richards JB (2018). Genetic architecture: the shape of the genetic contribution to human traits and disease. Nat Rev Genet.

[11] Farh KK-H, Marson A, Zhu J, Kleinewietfeld M, Housley WJ, Beik S (2015). Genetic and Epigenetic Fine-Mapping of Causal Autoimmune Disease Variants. Nature..

[12] Loh P-R, Bhatia G, Gusev A, Finucane HK, Bulik-Sullivan BK, Pollack SJ (2015). Contrasting genetic architectures of schizophrenia and other complex diseases using fast variance-components analysis. Nat Genet.

[13] Shi H, Kichaev G, Pasaniuc B (2016). Contrasting the genetic architecture of 30 complex traits from summary association data. Am J Hum Genet.

[14] Boyle EA, Li YI, Pritchard JK (2017). An expanded view of complex traits: from polygenic to omnigenic. Cell..

[15] Battle A, Mostafavi S, Zhu X, Potash JB, Weissman MM, McCormick C (2014). Characterizing the genetic basis of transcriptome diversity through RNA-sequencing of 922 individuals. Genome Res.

[16] Mähler N, Wang J, Terebieniec BK, Ingvarsson PK, Street NR, Hvidsten TR (2017). Gene co-expression network connectivity is an important determinant of selective constraint. PLoS Genet.

[17] Josephs EB, Wright SI, Stinchcombe JR, Schoen DJ (2017). The relationship between selection, network connectivity, and regulatory variation within a population of Capsella grandiflora. Genome Biol Evol.

[18] Barbeira AN, Dickinson SP, Bonazzola R, Zheng J, Wheeler HE, Torres JM (2018). Exploring the phenotypic consequences of tissue specific gene expression variation inferred from GWAS summary statistics. Nat Commun.

[19] Lonsdale J, Thomas J, Salvatore M, Phillips R, Lo E, Shad S (2013). The genotype-tissue expression (GTEx) project. Nat Genet.

[20] Langfelder P, Horvath S (2008). WGCNA: an R package for weighted correlation network analysis. BMC Bioinformatics.

[21] Zhu J, Lum PY, Lamb J, GuhaThakurta D, Edwards SW, Thieringer R (2004). An integrative genomics approach to the reconstruction of gene networks in segregating populations. Cytogenet Genome Res.

[22] Gusev A, Ko A, Shi H, Bhatia G, Chung W, Penninx BWJH (2016). Integrative approaches for large-scale transcriptome-wide association studies. Nat Genet.

[23] Liu JZ, Mcrae AF, Nyholt DR, Medland SE, Wray NR, Brown KM (2010). A versatile gene-based test for genome-wide association studies. Am J Hum Genet.

[24] Lamparter D, Marbach D, Rueedi R, Kutalik Z, Bergmann S (2016). Fast and rigorous computation of gene and pathway scores from SNP-based summary statistics. PLoS Comput Biol.

[25] Zhu X, Stephens M (2018). Large-scale genome-wide enrichment analyses identify new trait-associated genes and pathways across 31 human phenotypes. Nat Commun.

[26] Giambartolomei C, Vukcevic D, Schadt EE, Franke L, Hingorani AD, Wallace C (2014). Bayesian test for colocalisation between pairs of genetic association studies using summary statistics. PLoS Genet.

[27] Zhu Z, Zhang F, Hu H, Bakshi A, Robinson MR, Powell JE (2016). Integration of summary data from GWAS and eQTL studies predicts complex trait gene targets. Nat Genet.

[28] Hill SM, Heiser LM, Cokelaer T, Unger M, Nesser NK, Carlin DE (2016). Inferring causal molecular networks: empirical assessment through a community-based effort. Nat Methods.

[29] Zhu J, Zhang B, Smith EN, Drees B, Brem RB, Kruglyak L (2008). Integrating large-scale functional genomic data to dissect the complexity of yeast regulatory networks. Nat Genet.

[30] Divaraniya AA (2017). Mapping the Shared Molecular Architecture of Complex Inflammatory Diseases.

[31] Cohain A, Divaraniya AA, Zhu K, Scarpa JR, Kasarskis A, Zhu J (2017). Exploring the reproducibility of probabilistic causal molecular network models. Pacific Symposium on Biocomputing. World Scientific.

[32] ConsortiumT 1000 GP (2015). A global reference for human genetic variation. Nature.

[33] Blake JA, Richardson JE, Bult CJ, Kadin JA, Eppig JT (2003). MGD: the mouse genome database. Nucleic Acids Res.

[34] Duan Q, Reid SP, Clark NR, Wang Z, Fernandez NF, Rouillard AD (2016). L1000CDS2: LINCS L1000 characteristic direction signatures search engine. NPJ Syst Biol Appl.

[35] Wang Z, Monteiro CD, Jagodnik KM, Fernandez NF, Gundersen GW, Rouillard AD (2016). Extraction and analysis of signatures from the Gene Expression Omnibus by the crowd. Nat Commun.

[36] Clark NR, Hu KS, Feldmann AS, Kou Y, Chen EY, Duan Q (2014). The characteristic direction: a geometrical approach to identify differentially expressed genes. BMC Bioinformatics.

[37] Stuart JM, Segal E, Koller D, Kim SK (2003). A gene-coexpression network for global discovery of conserved genetic modules. Science (80- ).

[38] Gamazon ER, Wheeler HE, Shah KP, Mozaffari SV, Aquino-Michaels K, Carroll RJ (2015). A gene-based association method for mapping traits using reference transcriptome data. Nat Genet.

[39] Astle WJ, Elding H, Jiang T, Allen D, Ruklisa D, Mann AL (2016). The allelic landscape of human blood cell trait variation and links to common complex disease. Cell..

[40] Shoemaker JE, Lopes TJS, Ghosh S, Matsuoka Y, Kawaoka Y, Kitano H (2012). CTen: a web-based platform for identifying enriched cell types from heterogeneous microarray data. BMC Genomics.

[41] Jostins L, Ripke S, Weersma RK, Duerr RH, McGovern DP, Hui KY (2012). Host-microbe interactions have shaped the genetic architecture of inflammatory bowel disease. Nature..

[42] Cuzzocrea S, Rossi A, Mazzon E, Di Paola R, Genovese T, Muia C (2005). 5-Lipoxygenase modulates colitis through the regulation of adhesion molecule expression and neutrophil migration. Lab Investig.

[43] Jiao H, Rutar M, Fernando N, Yednock T, Sankaranarayanan S, Aggio-Bruce R (2018). Subretinal macrophages produce classical complement activator C1q leading to the progression of focal retinal degeneration. Mol Neurodegener.

[44] Sanyal R, Polyak MJ, Zuccolo J, Puri M, Deng L, Roberts L (2017). MS4A4A: a novel cell surface marker for M2 macrophages and plasma cells. Immunol Cell Biol.

[45] Mattiola I, Tomay F, De Pizzol M, Silva-Gomes R, Savino B, Gulic T (2019). The macrophage tetraspan MS4A4A enhances dectin-1-dependent NK cell–mediated resistance to metastasis. Nat Immunol.

[46] Charbonneau M, Lavoie RR, Lauzier A, Harper K, McDonald PP, Dubois CM (2016). Platelet-derived growth factor receptor activation promotes the prodestructive invadosome-forming phenotype of synoviocytes from patients with rheumatoid arthritis. J Immunol.

[47] Sandler C, Joutsiniemi S, Lindstedt KA, Juutilainen T, Kovanen PT, Eklund KK (2006). Imatinib mesylate inhibits platelet derived growth factor stimulated proliferation of rheumatoid synovial fibroblasts. Biochem Biophys Res Commun.

[48] Miyabe Y, Miyabe C, Iwai Y, Takayasu A, Fukuda S, Yokoyama W (2013). Necessity of lysophosphatidic acid receptor 1 for development of arthritis. Arthritis Rheum.

[49] Jin L, Zuo X-Y, Su W-Y, Zhao X-L, Yuan M-Q, Han L-Z (2014). Pathway-based analysis tools for complex diseases: a review. Genomics Proteomics Bioinformatics.

[50] Pierson E, Koller D, Battle A, Mostafavi S, ConsortiumGte (2015). Sharing and specificity of co-expression networks across 35 human tissues. PLoS Comput Biol.

[51] Califano A, Butte AJ, Friend S, Ideker T, Schadt E (2012). Leveraging models of cell regulation and GWAS data in integrative network-based association studies. Nat Genet.

[52] Yugi K, Kubota H, Hatano A, Kuroda S (2016). Trans-omics: how to reconstruct biochemical networks across multiple ‘omic’layers. Trends Biotechnol.

